# Open for business

**DOI:** 10.1038/sdata.2017.58

**Published:** 2017-06-13

To a researcher, open science may mean the freedom to access a paper without hitting a paywall. To an educator, it may mean the freedom to use figures in their class materials. To a start-up company, it may mean the ability to incorporate research results into their goods and services without negotiating complicated licences. This last use case—generating economic value through commercial enterprise—is a key aspect of the open science movement, and perhaps the most radical. It rests on a belief that the products of publicly funded research should be open for all to see—*and to use*. It is radical specifically because it requires that publishers, researchers and institutions forego traditional sources of revenue derived from licensing research outputs—whether through subscriptions or by licensing patents or databases. But, the potential benefit for society and the economy are clear. Open release of data from the human genome project and US Landsat satellite imagery have each been estimated to have generated billions, if not trillions of dollars in new economic value^1–3^.

*Scientific Data* is, however, routinely approached by prospective authors looking to publish descriptions of datasets that have restrictions on commercial use. As a journal dedicated to promoting open data, *Scientific Data* feels this is inconsistent with our aims and historically we have declined submissions of this kind. Our readers should feel confident that they can reuse the data described in our publications without needing to sift through details of restrictive licences or data use agreements.

This position is consistent with an influential community-developed definition of open data, which states that, ‘open data is data that can be freely used, re-used and redistributed by anyone—subject only, at most, to the requirement to attribute and sharealike’ (http://go.nature.com/2mSOhYg).

Today we are re-affirming our commitment to this policy. With the exception of sensitive datasets derived from humans, the journal will not consider submissions describing datasets with restrictions on commercial reuse, including those covered by the Creative Commons CC BY-NC licence (http://go.nature.com/2oeNmlh). Authors who have used data from commercial third-party sources when generating their own dataset will be asked to negotiate the right to release their data openly before submitting to the journal.

In addition, we will no longer offer the CC BY-NC licence as an option for our publications. We already use the CC BY licence as our default publishing licence (http://go.nature.com/2mSP6Au), and have used CC BY-NC only rarely at the journal. Going forward this option will no longer be available to our authors, without exception.

We understand that researchers may wish to restrict commercial use of their data for various reasons—not least of which being the desire to derive revenue from commercial licensing agreements. Researchers may also simply wish to retain some control over how their data are exploited in commercial settings. For researchers whose funding is partly or wholly derived from private for-profit sources, open data release may be incompatible with the business aims of their funder. These are all valid reasons, but we feel that data carrying commercial restrictions are simply not appropriate for publication in an open data journal like *Scientific Data*.

Commercial restrictions can also make data harder to use in academic research settings, in particular, by making them hard to mix and integrate with data that have less restrictive licences. Such data can fail at being ‘interoperable’, a key aspect of the FAIR Data Principles^4^. Furthermore, factual data are generally not protected by copyright, so whether non-commercial restrictions can even be enforced on research datasets is a complicated question that will depend on the nature of the data and local laws (in Europe and some other countries reusers must also contend with separate *sui generis* rights that apply to certain kinds of databases). Overall, releasing data under a non-commercial licence creates substantial uncertainty about how and where it can be reused, without actually granting the data sharer any ironclad control over the commercial use of the data (see also refs 5,^6^).

We therefore strongly encourage our authors to share their data under the Creative Commons CC0 waiver, a universal public domain declaration that frees research from any legal encumbrances. The CC0 waiver is automatically applied to all data uploaded to Dryad and figshare alongside a Data Descriptor, when using our integrated submission system. CC0 is well-suited for sharing research data alongside publications, and its use has been endorsed by a number of organizations (ref. 7 and http://go.nature.com/2nDKuwJ).
